# Using Implementation Science to Understand Teledermatology Implementation Early in the COVID-19 Pandemic: Cross-sectional Study

**DOI:** 10.2196/33833

**Published:** 2022-06-09

**Authors:** Shanelle Mariah Briggs, Jules Benjamin Lipoff, Sigrid Marie Collier

**Affiliations:** 1 School of Medicine University of Washington Seattle, WA United States; 2 Department of Dermatology University of Pennsylvania Philadelphia, PA United States; 3 Division of Dermatology University of Washington Seattle, WA United States

**Keywords:** teledermatology, telemedicine, telehealth, COVID-19, remote care, implementation science, store and forward, Organizational Readiness to Change Assessment, acceptability, feasibility, digital health, dermatology, dermatologist, health technology

## Abstract

**Background:**

Implementation science has been recognized for its potential to improve the integration of evidence-based practices into routine dermatologic care. The COVID-19 pandemic has resulted in rapid teledermatology implementation worldwide. Although several studies have highlighted patient and care provider satisfaction with teledermatology during the COVID-19 pandemic, less is known about the implementation process.

**Objective:**

Our goal was to use validated tools from implementation science to develop a deeper understanding of the implementation of teledermatology during the COVID-19 pandemic. Our primary aims were to describe (1) the acceptability and feasibility of the implementation of teledermatology and (2) organizational readiness for the implementation of teledermatology during the COVID-19 pandemic. We also sought to offer an example of how implementation science can be used in dermatologic research.

**Methods:**

An anonymous, web-based survey was distributed to Association of Professors of Dermatology members. It focused on (1) the acceptability, feasibility, and appropriateness of teledermatology and (2) organizational readiness for implementing teledermatology. It incorporated subscales from the Organizational Readiness to Change Assessment—a validated measure of organizational characteristics that predict implementation success.

**Results:**

Of the 518 dermatologists emailed, 35 (7%) responded, and all implemented or scaled up teledermatology during the pandemic. Of the 11 care providers with the highest level of organizational readiness, 11 (100%) said that they plan to continue using teledermatology after the pandemic. Most respondents agreed or strongly agreed that they had sufficient training (24/35, 69%), financial resources (20/35, 57%), and facilities (20/35, 57%). However, of the 35 respondents, only 15 (43%) agreed or strongly agreed that they had adequate staffing support. Most respondents considered the most acceptable teledermatology modality to be synchronous audio and video visits with supplemental stored digital photos (23/35, 66%) and considered the least acceptable modality to be telephone visits without stored digital photos (6/35, 17%). Overall, most respondents thought that the implementation of synchronous audio and video with stored digital photos (31/35, 89%) and telephone visits with stored digital photos (31/35, 89%) were the most feasible. When asked about types of visits that were acceptable for synchronous video/audio visits (with stored digital photos), 18 of the 31 respondents (58%) said “new patients,” 27 (87%) said “existing patients,” 19 (61%) said “medication monitoring,” 3 (10%) said “total body skin exams,” and 22 (71%) said “lesions of concern.”

**Conclusions:**

This study serves as an introduction to how implementation science research methods can be used to understand the implementation of novel technologies in dermatology. Our work builds upon prior studies by further characterizing the acceptability and feasibility of different teledermatology modalities. Our study may suggest initial insights on how dermatology practices and health care systems can support dermatologists in successfully incorporating teledermatology after the pandemic.

## Introduction

When the COVID-19 pandemic began in the spring of 2020, restrictions were placed on in-person visits. This crisis led to the rapid increase in teledermatology implementation, which was made possible by policy changes that overcame prior barriers to implementation, such as the lack of insurance reimbursement, liability concerns, and licensing restrictions [1,2]. A recent review highlighted teledermatology’s potential to reduce health care disparities in underserved and marginalized communities, calling for future efforts to study implementation, as teledermatology has expanded greatly during the pandemic [3]. Although much of the published work on teledermatology during the COVID-19 pandemic focused on satisfaction among patients and care providers, less is known about its actual implementation during the early months of the pandemic [4,5]. The field of implementation science has been recognized for its potential to improve the integration of evidence-based practices into routine dermatologic care [6].

In order for teledermatology to be successfully incorporated into routine dermatologic practice, there must be buy-in from dermatology patients, dermatologists, and health systems. Our study adds to the growing body of evidence for teledermatology by using validated implementation science tools to develop a deeper understanding of the implementation of teledermatology during the COVID-19 pandemic.

Implementation science uses specific terminology to describe key predictors of and outcomes for the implementation of evidence-based interventions. Implementation outcomes refer to “the effects of deliberate and purposive actions to implement new treatments, practices, and services,” and they “[serve] as an indicator of implementation success” [7]. In this study, we focused on evaluating the *acceptability* and *feasibility* of teledermatology, which are defined, respectively, as follows: (1) “the perception among implementation stakeholders that a given treatment, service, practice, or innovation is agreeable, palatable, or satisfactory” and (2) “the extent to which a new treatment, or innovation, can be successfully used or carried out within a given agency or setting” [7]. We also evaluated *organizational readiness* for change, which is defined as “the extent to which organizational members are psychologically and behaviourally prepared to implement organizational change” [8].

We use the lens of implementation science to describe teledermatology implementation. The objective of our study was to understand the acceptability and feasibility of the implementation of teledermatology during the COVID-19 pandemic, as well as organizational readiness for the implementation of teledermatology. We also sought to offer an example of how implementation science can be used in dermatologic research.

## Methods

### Ethical Considerations

Our study was deemed exempt from ethics approval by the University of Washington Human Subjects Division and the University of Washington Institutional Review Board (IRB ID: STUDY00010266).

### Study Design

We conducted a cross-sectional study of dermatologists’ perceptions of teledermatology implementation during the COVID-19 pandemic. We surveyed members of the Association of Professors of Dermatology (APD) between November 20 and December 9, 2020 (Multimedia Appendix 1). An initial email was sent on November 20, and it was resent on December 2 to try to increase the response rate. The survey focused on (1) the acceptability, feasibility, and appropriateness of teledermatology and (2) organizational readiness for implementing teledermatology. We used an abbreviated, single-item version of a validated scale [9] to assess the acceptability of different teledermatology modalities (eg, synchronous audio and video and stored digital photos). Using the same scale, we assessed the acceptability of teledermatology for different dermatologic conditions and purposes (eg, lesions of concern and medication monitoring). We also incorporated selected subscales from the validated Organizational Readiness to Change Assessment (ORCA) instrument (eg, culture and resources). There were 26 ORCA questions, which were scored on a 5-point Likert scale ranging from “Strongly Disagree” to “Strongly Agree.” The possible ORCA scores ranged from 26 to 130, with higher scores indicating higher organizational readiness for change. The entire survey was tested for face validity and readability through pilots with dermatologists. It was iteratively refined based on their feedback. The survey was administered via email, and responses were collected anonymously. ORCA scores were reported as unweighed composite scores, and participants were stratified by ORCA score tertiles—“low,” “medium,” and “high” organizational readiness for change (Table 1). We did not perform statistical hypothesis testing, in accordance with best practices [10].

**Table 1:** Demographics and outcomes by Organizational Readiness to Change Assessment (ORCA) tertiles.

		ORCA score tertiles				Total (N=35)
		Low (n=11)	Medium (n=13)	High (n=11)		
**Sex, n (%)**						
	Male	2 (18)	6 (47)	5 (46)	13 (37)	
	Female	9 (82)	7 (54)	6 (55)	22 (63)	
**Race, n (%)**						
	American Indian or Alaska Native	1 (9)	0 (0)	0 (0)	1 (3)	
	Asian	1 (9)	2 (15)	2 (18)	5 (14)	
	Black or African American	1 (9)	0 (0)	0 (0)	1 (3)	
	Hispanic, Latinx, or Spanish origin	1 (9)	2 (15)	1 (9)	4 (11)	
	White	7 (64)	9 (69)	8 (73)	24 (69)	
**Practice, n (%)**						
	Dermatology group practice	0 (0)	0 (0)	2 (18)	2 (6)	
	Multispecialty group practice	1 (9)	0 (0)	0 (0)	1 (3)	
	Academic practice	9 (82)	12 (92)	9 (82)	30 (86)	
	Veterans administration and academic practice	1 (9)	1 (8)	0 (0)	2 (6)	
**Number of years in practice**						
	Years, mean (SD)	3.91 (1.97)	3.69 (1.75)	3.82 (1.78)	3.80 (1.78)	
	Years, median (quartile 1, quartile 3)	4.00 (2.50, 6.00)	3.00 (2.00, 6.00)	4.00 (3.00, 5.00)	4.00 (2.00, 6.00)	
**Total ORCA score**						
	Score, mean (SD)	70.0 (9.58)	90.4 (5.36)	110.7 (8.75)	90.4 (18.1)	
	Score, median (quartile 1, quartile 3)	68.00 (66.0, 78.0)	91.0 (88.0, 96.0)	110.0 (104.0, 113.5)	91.0 (78.5, 102.5)	
**Stored digital photos alone are acceptable, n (%)**						
	Completely agree or agree	7 (64)	5 (39)	6 (55)	18 (51)	
	Completely disagree, disagree, or neither agree nor disagree	4 (36)	8 (62)	5 (46)	17 (49)	
**Telephone visits without photos are acceptable, n (%)**						
	Completely agree or agree	2 (18)	1 (8)	3 (27)	6 (17)	
	Completely disagree, disagree, or neither agree nor disagree	9 (82)	12 (92)	8 (73)	29 (83)	
**Telephone visits with photos are acceptable, n (%)**						
	Completely agree or agree	7 (64)	7 (54)	6 (55)	20 (57)	
	Completely disagree, disagree, or neither agree nor disagree	4 (36)	6 (46)	5 (46)	15 (43)	
**Synchronous audio and video visits without photos are acceptable, n (%)**						
	Completely agree or agree	3 (27)	4 (31)	5 (46)	12 (34)	
	Completely disagree, disagree, or neither agree nor disagree	8 (73)	9 (69)	6 (55)	23 (66)	
**Synchronous audio and video visits with photos are acceptable, n (%)**						
	Completely agree or agree	4 (36)	11 (85)	8 (73)	23 (66)	
	Completely disagree, disagree, or neither agree nor disagree	7 (64)	2 (15)	3 (27)	12 (34)	
**Plan to use telemedicine after the pandemic, n (%)**						
	Yes	10 (91)	12 (92)	11 (100)	33 (94)	
	No	1 (9)	1 (8)	0 (0)	2 (6)	

## Results

Of the 518 dermatologists on the APD email listserv, 35 (7%) responded, and all implemented or scaled up teledermatology during the pandemic. Of the 35 respondents, 35 (100%) said that the peak use of teledermatology occurred between the months of April and September 2020. Thus, all respondents had completed the initial implementation by the time of survey distribution in December 2020. Further, 94% (33/35) plan to continue using teledermatology after the pandemic. The benefits of teledermatology included less travel time and expense for patients (n=35, 100%), continued patient care (n=33, 94%), the ability to avoid the risk of infection (n=35, 100%), and work flexibility (n=27, 77%). Respondents also experienced challenges with teledermatology, including technology issues (n=22, 63%) and challenges with caring for older adults (n=18, 51%). All 11 care providers with “high” ORCA scores said that they plan to continue using teledermatology after the pandemic (Table 1). With regard to organizational readiness for the implementation of teledermatology during the pandemic, 24 of the 35 care providers (69%) agreed or strongly agreed that they had sufficient training, 20 (57%) had sufficient financial resources, and 20 (57%) had sufficient facilities. Most respondents had care provider buy-in (25/35, 71%) and felt that teledermatology implementation took into consideration the needs and preferences of patients (27/35, 77%). On the other hand, fewer respondents had a dedicated team for implementing the intervention (14/34, 41%), had sufficient staffing support (15/35, 43%), or had successfully piloted telemedicine prior to the pandemic (13/35, 37%). Most of the 35 respondents reported using several implementation strategies, which included a dedicated clinical champion (n=26, 74%); feedback to clinicians (n=20, 59%); education (n=24, 69%); and, less commonly, staff incentives (n=4, 11%). Table S1 in Multimedia Appendix 2 shows these supplemental results. Of the 11 respondents with “low” ORCA scores, 6 (55%) agreed or strongly agreed with the ORCA components about having a clinical champion, 5 (45%) agreed or strongly agreed with giving feedback to clinicians, 2 (18%) agreed or strongly agreed with education, and 0 (0%) agreed or strongly agreed with staff incentives. Of the 11 participants with “high” ORCA scores, 11 (100%) agreed or strongly agreed with having a clinical champion, 9 (82%) agreed or strongly agreed with giving feedback to clinicians, 10 (91%) agreed or strongly agreed with education, and 4 (36%) agreed or strongly agreed with staff incentives (Table S2 in Multimedia Appendix 2).

The most acceptable teledermatology modality was synchronous audio and video visits with stored digital photos (23/35, 66%). The least acceptable modality was telephone visits without stored digital photos (6/35, 17%). When comparing participants with “low” ORCA scores to those with “medium” and “high” ORCA scores, synchronous audio and video visits with stored digital photos were less acceptable among those with “low” ORCA scores (4/11, 36%) relative to those with “medium” (11/13, 85%) and “high” (8/11, 73%) ORCA scores. However, the acceptability of consultations involving stored digital photos was higher among those with “low” ORCA scores (7/11, 64%) relative to those with “medium” (5/13, 39%) and “high” (6/11, 55%) ORCA scores. Along with acceptability (Table 1), feasibility was also addressed. Overall, among the 35 respondents, synchronous audio and video visits with stored digital photos (n=31, 89%) and telephone visits with stored digital photos (n=31, 89%) were deemed the most feasible teledermatology modalities. Other modalities were also deemed feasible, though less so, including consultations involving stored digital photos (n=26, 74%), synchronous audio and video visits without stored digital photos (n=23, 66%), and telephone visits without stored digital photos (n=21, 60%). When asked about types of visits that were acceptable for synchronous video/audio visits (with stored digital photos), 18 of the 31 respondents (58%) said “new patients,” 27 (87%) said “existing patients,” 19 (61%) said “medication monitoring,” 3 (10%) said “total body skin exams,” and 22 (71%) said “lesions of concern. The majority of surveyed dermatologists felt that synchronous video/audio (without stored digital photos was acceptable for “existing patients” (29/32, 91%) and “medication monitoring” (29/32, 91%). Fewer respondents felt that synchronous video/audio (without stored digital photos) was acceptable for “new patients” (12/32, 38%), and very few felt that teledermatology was acceptable for “lesions of concern” (5/32, 16%) and “total body skin exams” (2/32, 6%).. Additional detailed results are shown in Table 1.

## Discussion

### Principal Results

In our study, although most dermatologists (33/35, 94%) planned to continue using teledermatology after the pandemic, there was some indication that they lacked support in certain areas (eg, staffing and facilities) during implementation early in the COVID-19 pandemic. Additionally, not all teledermatology modalities were equally acceptable or feasible. Among respondents, telephone and synchronous audio and video visits were the least acceptable and feasible modalities, whereas modalities that combined stored digital photos with telephone visits or synchronous audio and video visits were the most acceptable and feasible modalities.

Teledermatology has been a part of dermatologic care for over 25 years [11]. Although consultations involving stored digital photos (store and forward) have historically been the dominant teledermatology modality in clinical practice [12], the COVID-19 pandemic has resulted in the rapid implementation of synchronous audio and video teledermatology [13].

Although the majority of dermatologists (33/35, 94%) in this study planned to continue using teledermatology after the pandemic, there is likely some variability in intentions to continue using teledermatology, depending on the population surveyed and timing. An earlier survey by the American Academy of Dermatology in May 2020, which included a larger proportion of private practice dermatologists, found that just over half (58%) of dermatologists planned to continue using teledermatology after the pandemic [13]. Despite the differences, both surveys highlight the importance of teledermatology in the future, with over 50% of dermatologists intending to practice teledermatology.

Overall, respondents supported the use of teledermatology after the pandemic; however, we found that both telephone visits and synchronous audio and video visits without stored digital photos were the two least acceptable and feasible modalities. This finding aligns with patients’ experiences with teledermatology. Despite high levels of patient satisfaction and willingness to continue using teledermatology after the pandemic [4,5], satisfaction with dermatology telephone visits is lower, and fewer dermatology patients are willing to use telephone visits for future dermatologic care [14]. Taken as a whole, our findings build on a growing body of evidence that certain modalities, particularly telephone visits, are less acceptable to both patients and care providers. Dermatologists rely upon the clear and accurate visualization of the skin, which telephone and synchronous video/audio visits alone may not offer.

Incorporating stored digital photos may overcome some of the limitations of using synchronous audio and video visits and telephone visits in isolation. Acceptability was higher for including stored digital photos with synchronous audio and video visits or telephone visits when compared to that for visits without stored digital photos and consultations involving stored digital photos alone. Prior studies that were conducted during the COVID-19 pandemic showed that care providers were split between preferring synchronous (54%) and asynchronous (46%) modalities but did not assess the combination of asynchronous and synchronous approaches [15]. Dermatologists may be able to maximize the benefits of synchronous modalities (synchronous audio and video visits and telephone visits) and asynchronous modalities (stored digital photos) by combining them to create a more acceptable and preferable teledermatology experience.

We also found that there may be variability in the acceptability of teledermatology based on the type of dermatologic condition and visit. The majority of surveyed dermatologists felt that synchronous video/audio (without stored digital photos) was acceptable for “existing patients” (29/32, 91%) and “medication monitoring” (29/32, 91%). Fewer respondents felt that synchronous video/audio (without stored digital photos) was acceptable for “new patients” (12/32, 38%), and very few felt that teledermatology was acceptable for “lesions of concern” (5/32, 16%) and “total body skin exams” (2/32, 6%). The American Academy of Dermatology’s survey found a similarly low number of dermatologists who were comfortable with performing total body skin exams via teledermatology, with 96% believing that this requires an in-person examination [13]. This work adds to our understanding of the types of patient concerns for which teledermatology is the most acceptable. We hope that teledermatology guidelines for best practices can evolve via this growing collection of work.

When implementing novel health care technologies (including teledermatology technologies), organizational factors, such as organizational readiness for change, are important determinants of implementation success [16]. We found that although support for teledermatology implementation was high in most areas (eg, training and care provider buy-in), dermatologists lacked organizational support in other areas, such as staffing support and facilities. In addition, we found that respondents with a “low” organizational readiness for change tended to find synchronous audio and video visits less acceptable when compared to respondents with “medium” and “high” ORCA scores. It is plausible that in dermatology practices with a lower organizational readiness for change, limited support for newly implementing synchronous audio and video teledermatology [13] resulted in negative experiences during the COVID-19 pandemic. This hints at the potential importance of organizational readiness in determining the success of teledermatology implementation. Although specific implementation process details were outside the scope of this work, lower scores for important implementation strategies, including having a clinical champion, giving feedback to clinicians, and providing education, contributed to respondents having “low” scores for organizational readiness for change, and this may provide clues as to the specific implementation strategies that are important for the successful implementation of synchronous audio and video teledermatology early in the COVID-19 pandemic. Future research will be needed to explore the roles of specific implementation strategies, implementation processes, and costs in determining the success of newly implemented teledermatology programs.

We acknowledge that this cross-sectional survey has significant limitations, given its modest sample size and response rate. Therefore, we cannot draw definitive conclusions on the associations between organizational readiness for change and teledermatology implementation outcomes. Still, our total of 35 respondents and response rate of 7% (35/518) are similar to those of other nonincentivized physician surveys [13]. For these reasons, the findings of this survey may not be generalizable. As the survey was distributed to the APD, the majority of respondents (32/35) practiced in academic dermatology settings. Thus, respondents with an interest in teledermatology may have been overrepresented. Additionally, respondents were mostly White (24/35, 69%); as such, the opinions of dermatologists from all backgrounds were not captured. Despite these limitations, this work provides valuable descriptive insights into the role of implementation science in understanding teledermatology implementation during the COVID-19 pandemic.

### Conclusions

This study serves as an introduction to how implementation science research methods can be used to understand the implementation of novel technologies in dermatology. Our work builds on prior work by further characterizing the acceptability and feasibility of different teledermatology modalities. Our study also contributes initial insights on how dermatology practices and health care systems can support dermatologists in successfully incorporating teledermatology after the pandemic. Finally, this work highlights newer methods for identifying organizational factors that can be optimized to improve future teledermatology implementation efforts.

## Appendix

Multimedia Appendix 1 Survey instrument and recruitment letter/email.

Multimedia Appendix 2 Supplemental results.

## Abbreviations

APD: Association of Professors of Dermatology
ORCA: Organizational Readiness to Change Assessment
