# Supplementary material for: Using Implementation Science to Understand Teledermatology Implementation Early in the COVID-19 Pandemic: Cross-sectional Study
Source: JMIR Dermatol. 2022 Jun 9;5(2):e33833. doi: 10.2196/33833 (PMC9191554; doi:10.2196/33833)
Supplement: Multimedia Appendix 1 [file derma_v5i2e33833_app1.docx]

Appendix 1: Survey Instrument and Recruitment letter/email.


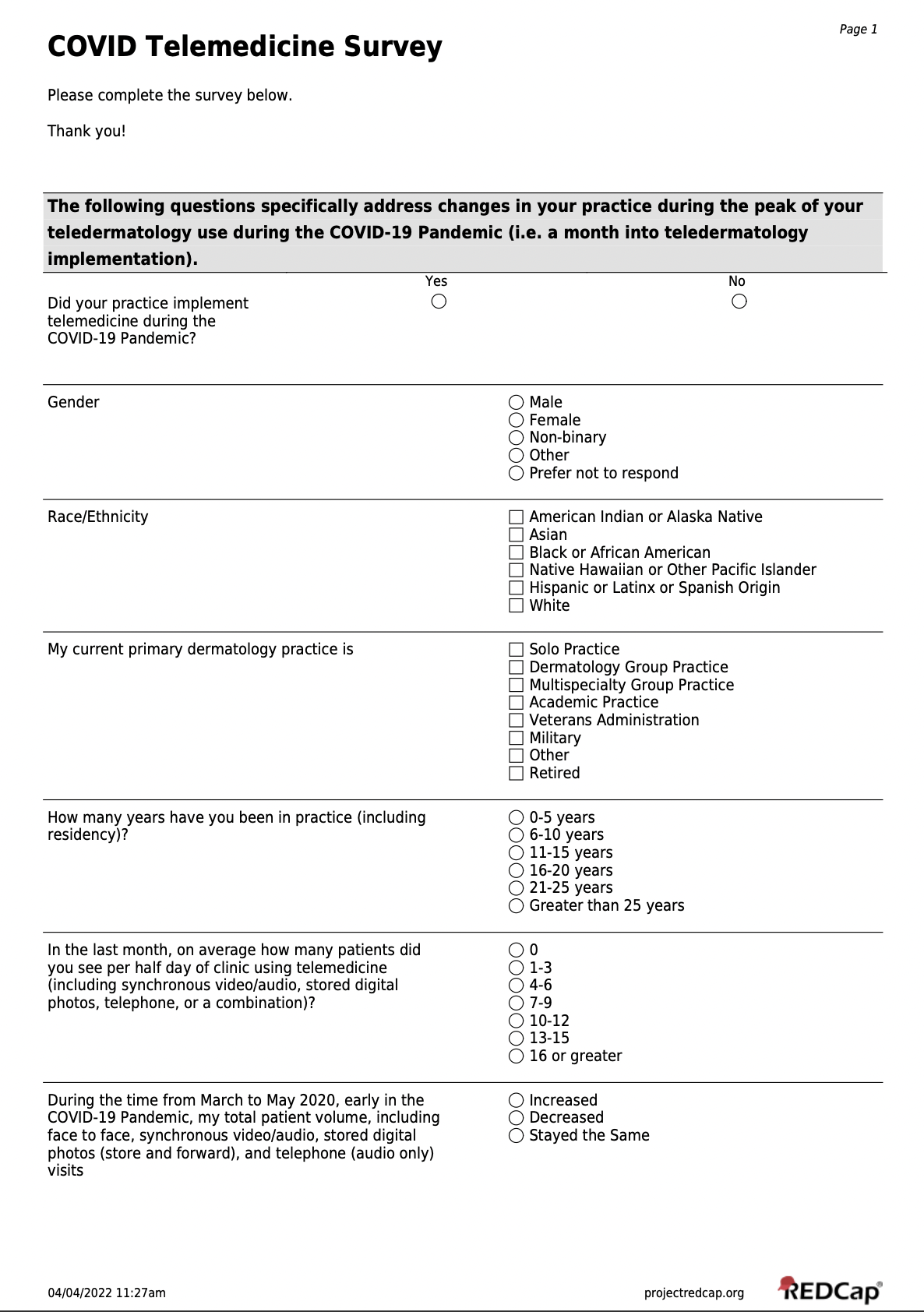


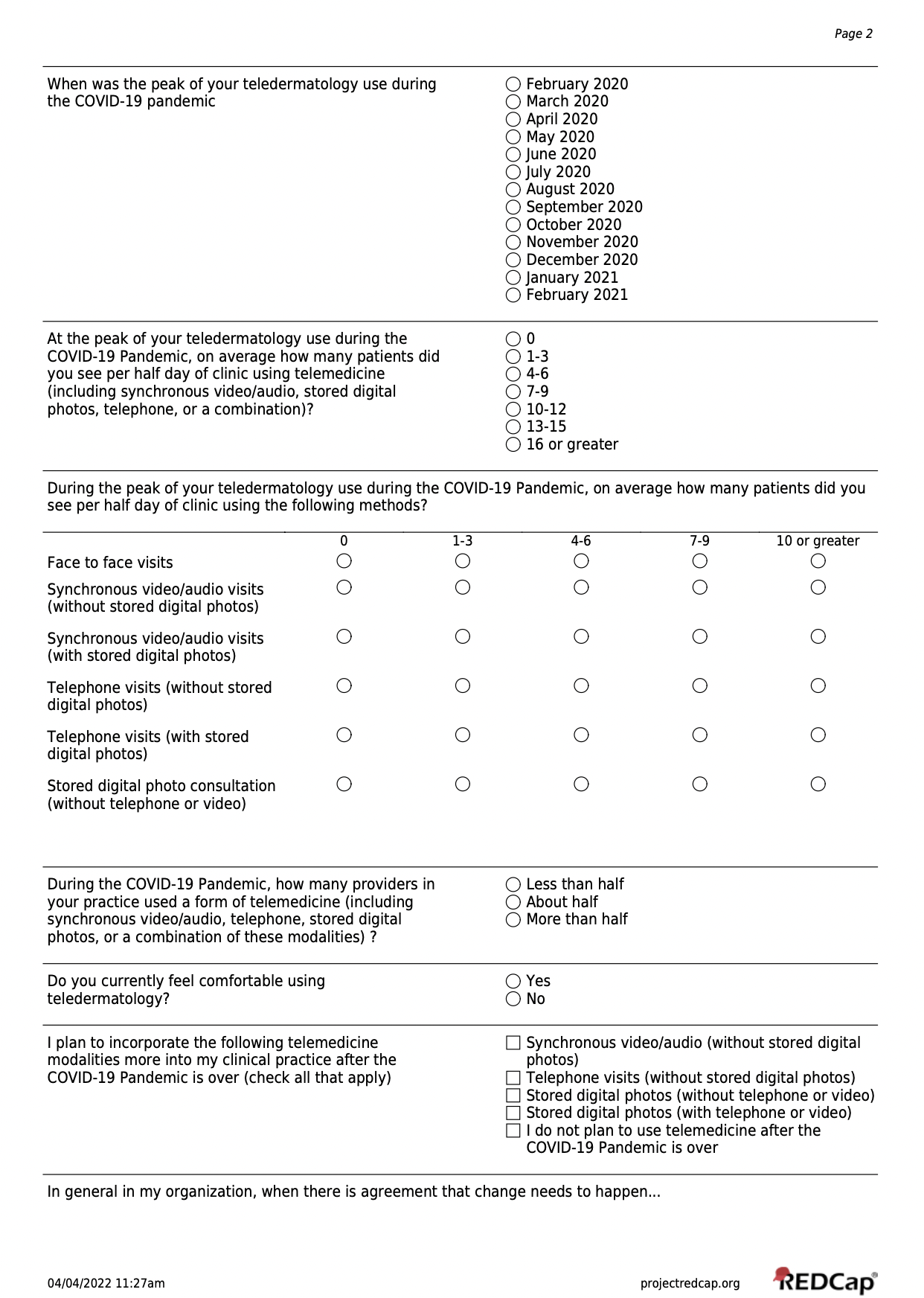


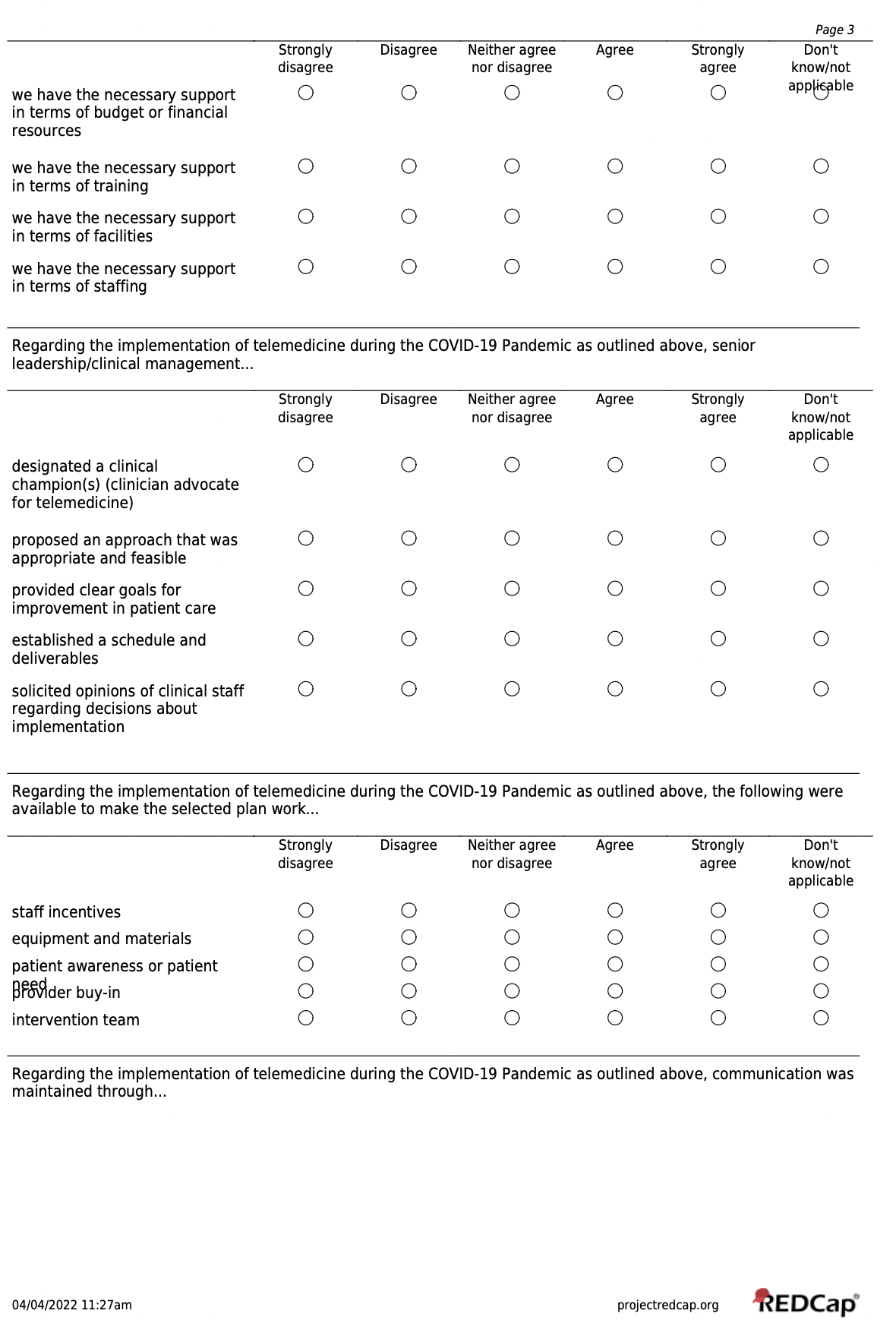


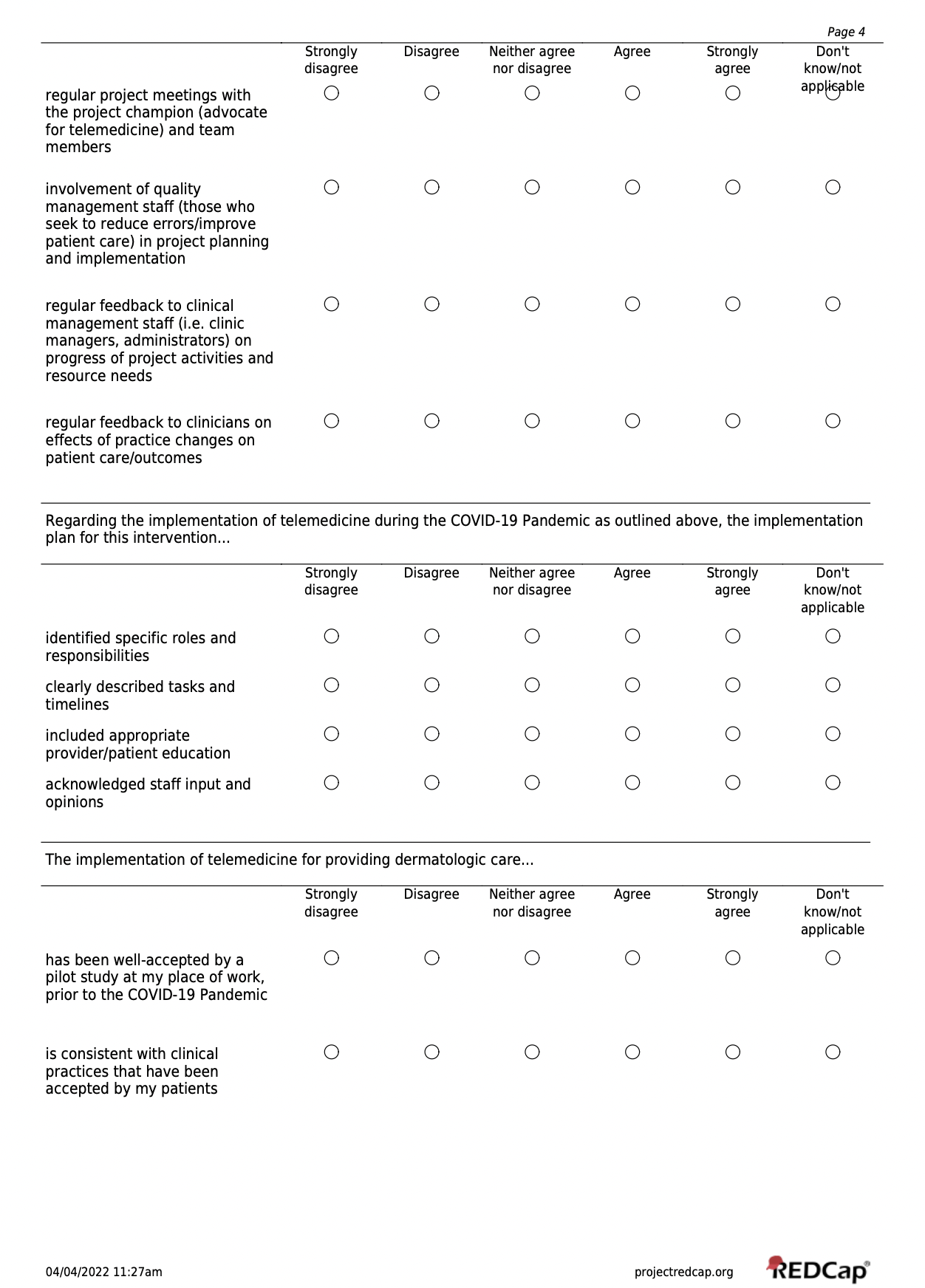


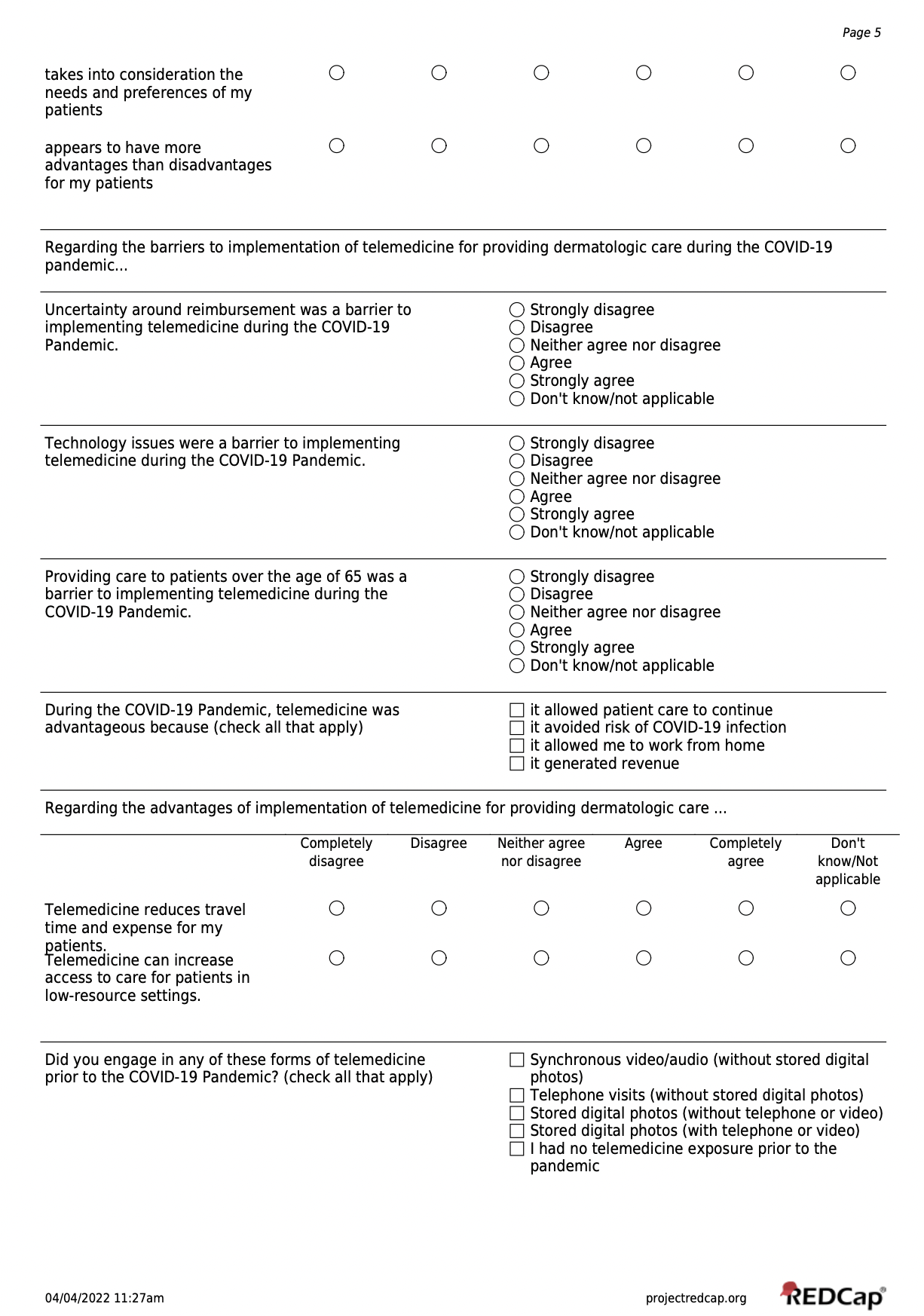


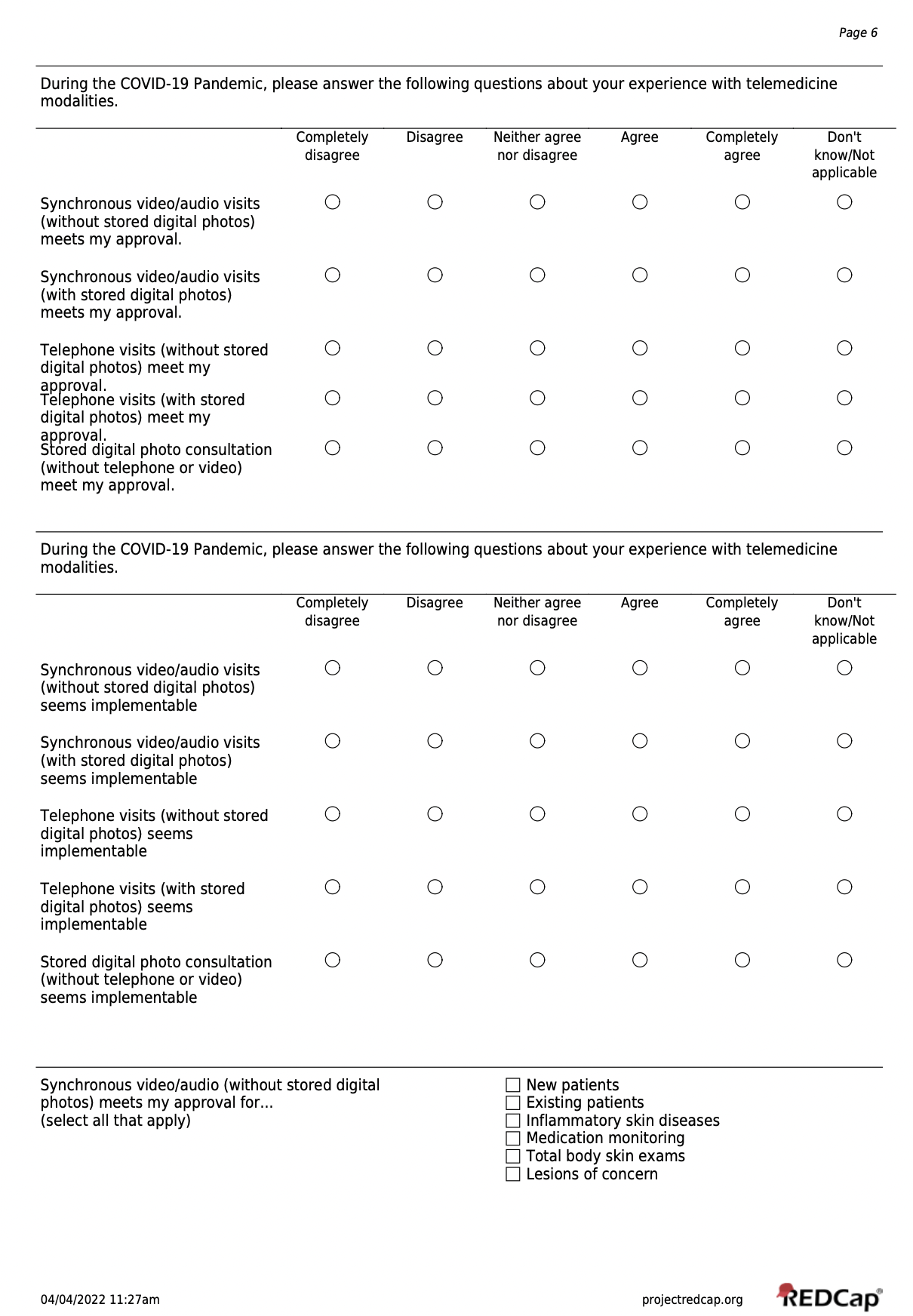


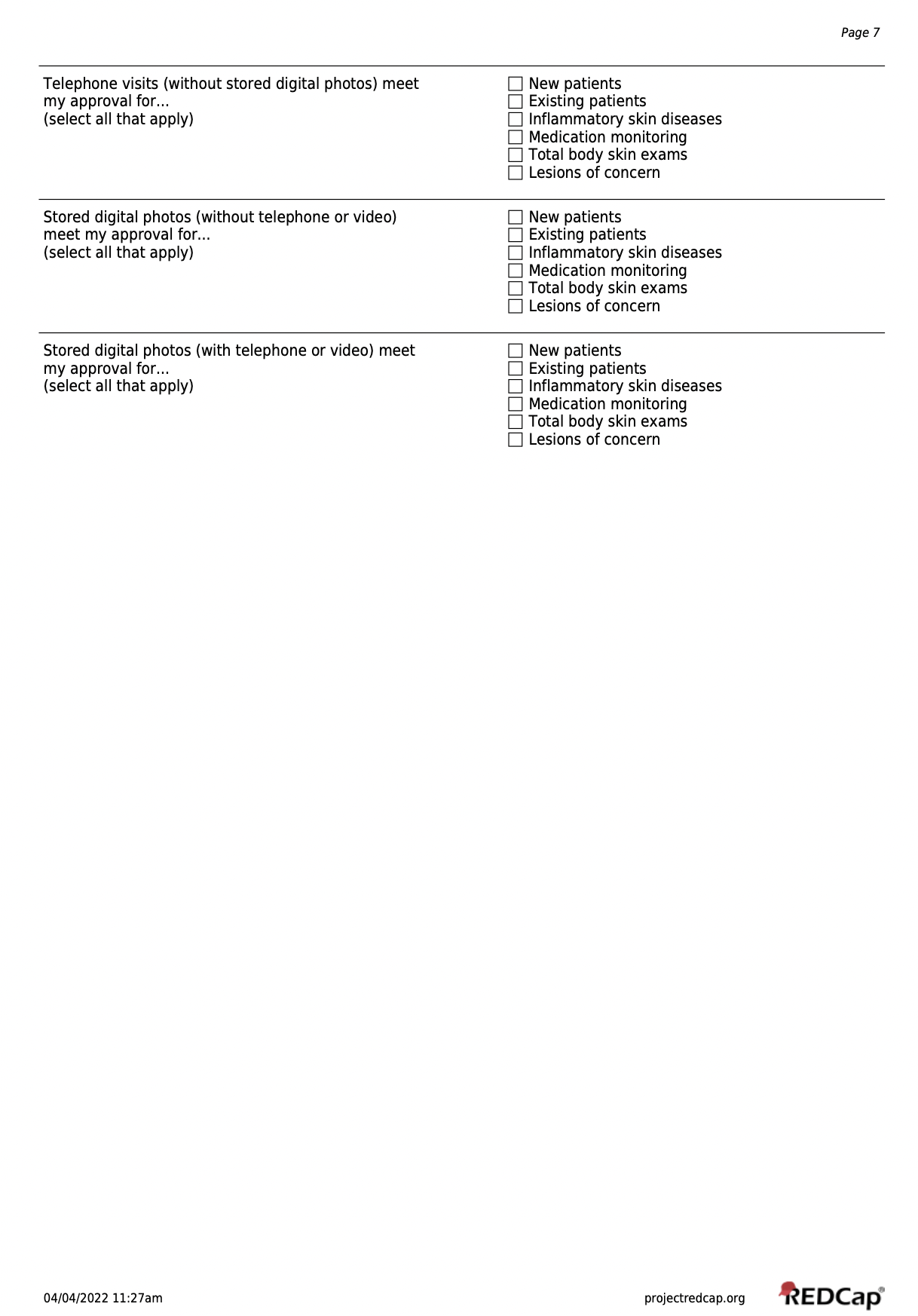


**Recruitment Letter:**

Sent on November 20^th^ and December 2^nd^ of 2020.

Subject:

From the University of Washington: We need YOUR input to understand teledermatology during the COVID-19 Pandemic.

Dear Association of Professors of Dermatology Member,

      I am reaching out to you because you likely used teledermatology during the COVID-19 Pandemic, and like many of us you had to quickly adapt to teledermatology in your practice. I would like to invite you to participate in a 5-10 minute survey about your experience with teledermatology during the COVID-19 Pandemic.

     We want to find out what factors helped (or hurt) the process of rapidly implementing or increasing teledermatology during the early months of the COVID-19 Pandemic. We plan to use your input to advance the field of dermatology! Your experiences will contribute to the development of best practices to make the teledermatology start-up process easier and more successful for other dermatologists both in the US and around the world.

We will provide the final survey results to the APD and will present the results at an APD-sponsored forum upon request. This study was reviewed and found to be exempt by the University of Washington IRB.

IRB study # 00010266

Your input is valuable. This will take 5-10 minutes, and we appreciate every minute of your time. You can take the survey here: https://redcap.iths.org/surveys/?s=LMW7MKT3PD

We look forward to hearing from you!

Sigrid Collier, MD, MPH University of Washington

Shanelle Briggs, MS3 University of Washington

Jules Lipoff, MD Penn Medicine

Please contact Shanelle Briggs at [briggsha@uw.edu](mailto:briggsha@uw.edu) with any questions.
