# Supplementary material for: Using Implementation Science to Understand Teledermatology Implementation Early in the COVID-19 Pandemic: Cross-sectional Study
Source: JMIR Dermatol. 2022 Jun 9;5(2):e33833. doi: 10.2196/33833 (PMC9191554; doi:10.2196/33833)
Supplement: Multimedia Appendix 2 [file derma_v5i2e33833_app2.docx]

Appendix 2: Supplemental Results.

Table S1: Supplemental data for Implementation Strategies from the adapted Organizational Readiness to Change Assessment (ORCA).

|  | Strongly Disagree or Disagree | Neither agree nor disagree | Strongly Agree or Agree | Don't know/not applicable |
| --- | --- | --- | --- | --- |
|  | n (%) | n (%) | n (%) | n (%) |
| **Regarding the implementation of telemedicine during the COVID-19 Pandemic as outlined above, senior leadership/clinical management...** |  |  |  |  |
| designated a clinical champion(s) | 5 (14%) | 4 (11%) | 26 (74%) | 0 (0%) |
| proposed an approach that was appropriate and feasible | 5 (14%) | 7 (20%) | 23 (66%) | 0 (0%) |
| provided clear goals for improvement in patient care | 8 (23%) | 6 (17%) | 21 (60%) | 0 (%) |
| established a schedule and deliverables | 8 (23%) | 6 (17%) | 21 (60%) | 0 (0%) |
| solicited opinions of clinical staff regarding decisions about implementation | 8 (23%) | 5 (14%) | 22 (63%) | 0 (0%) |
| **Regarding the implementation of telemedicine during the COVID-19 Pandemic as outlined above, the following were available to make the selected plan work...** |  |  |  |  |
| staff incentives | 24 (69%) | 6 (17%) | 4 (11%) | 1 (3%) |
| equipment and materials | 10 (29%) | 7 (20%) | 18 (51%) | 0 (0%) |
| patient awareness or patient need | 2 (6%) | 8 (23%) | 25 (71%) | 0 (0%) |
| provider buy-in | 4 (11%) | 6 (17%) | 25 (71%) | 0 (0%) |
| intervention team | 8 (24%) | 9 (26%) | 14 (41%) | 3 (9%) |
| **Regarding the implementation of telemedicine during the COVID-19 Pandemic as outlined above, communication was maintained through...** |  |  |  |  |
| regular project meetings with the project champion and team members | 8 (24%) | 6 (18%) | 19 (56%) | 1 (3%) |
| involvement of quality management staff in project planning and implementation | 11 (32%) | 7 (21%) | 14 (41%) | 2 (6%) |
| regular feedback to clinical management staff on progress of project activities and resource needs | 5 (15%) | 6 (18%) | 21 (62%) | 2 (6%) |
| regular feedback to clinicians on effects of practice changes on patient care/outcomes | 10 (29%) | 3 (9%) | 20 (59%) | 1 (3%) |
| **Regarding the implementation of telemedicine during the COVID-19 Pandemic as outlined above, the implementation plan for this intervention...** |  |  |  |  |
| identified specific roles and responsibilities | 5 (14%) | 7 (20%) | 23 (66%) | 0 (0%) |
| clearly described tasks and timelines | 4 (11%) | 10 (29%) | 21 (60%) | 0 (0%) |
| included appropriate provider/patient education | 5 (14%) | 6 (17%) | 24 (69%) | 0 (0%) |
| acknowledged staff input and opinions | 5 (15%) | 10 (29%) | 19 (56%) | 0 (0%) |

Legend: This table details the self-reported use of these implementation strategies among the respondents.

Table S2: Supplemental data for Implementation Strategies by ORCA tertile.

|  | **ORCA Tertiles** | | | |
| --- | --- | --- | --- | --- |
|  | Low (n=11) | Medium (n=13) | High (n=11) | Total (N=35) |
| **Regarding the implementation of telemedicine during the COVID-19 Pandemic as outlined above, senior leadership/clinical management...** | | | | |
| **designated a clinical champion(s) (clinician advocate for telemedicine)** |  |  |  |  |
| Strongly disagree | 1 (9%) | 0 (0%) | 0 (0%) | 1 (3%) |
| Disagree | 4 (36%) | 0 (0%) | 0 (0%) | 4 (11%) |
| Neither agree nor disagree | 0 (0%) | 4 (31%) | 0 (0%) | 4 (11%) |
| Agree | 4 (36%) | 5 (38%) | 5 (45%) | 14 (40%) |
| Strongly agree | 2 (18%) | 4 (31%) | 6 (55%) | 12 (34%) |
| Don’t know/not applicable | 0 (0%) | 0 (0%) | 0 (0%) | 0 (0%) |
| **proposed an approach that was appropriate and feasible** |  |  |  |  |
| Strongly disagree | 1 (9%) | 0 (0%) | 0 (0%) | 1 (3%) |
| Disagree | 4 (36%) | 0 (0%) | 0 (0%) | 4 (11%) |
| Neither agree nor disagree | 4 (36%) | 3 (23%) | 0 (0%) | 7 (20%) |
| Agree | 2 (18%) | 6 (46%) | 5 (45%) | 13 (37%) |
| Strongly agree | 0 (0%) | 4 (31%) | 6 (55%) | 10 (29%) |
| Don’t know/not applicable | 0 (0%) | 0 (0%) | 0 (0%) | 0 (0%) |
| **provided clear goals for improvement in patient care** |  |  |  |  |
| Strongly disagree | 1 (9%) | 0 (0%) | 0 (0%) | 1 (3%) |
| Disagree | 6 (55%) | 1 (8%) | 0 (0%) | 7 (20%) |
| Neither agree nor disagree | 1 (9%) | 5 (38%) | 0 (0%) | 6 (17%) |
| Agree | 3 (27%) | 5 (38%) | 8 (73%) | 16 (46%) |
| Strongly agree | 0 (0%) | 2 (15%) | 3 (27%) | 5 (14%) |
| Don’t know/not applicable | 0 (0%) | 0 (0%) | 0 (0%) | 0 (0%) |
| **established a schedule and deliverables** |  |  |  |  |
| Strongly disagree | 2 (18%) | 0 (0%) | 0 (0%) | 2 (6%) |
| Disagree | 5 (45%) | 1 (8%) | 0 (0%) | 6 (17%) |
| Neither agree nor disagree | 3 (27%) | 3 (23%) | 0 (0%) | 6 (17%) |
| Agree | 1 (9%) | 7 (54%) | 7 (64%) | 15 (43%) |
| Strongly agree | 0 (0%) | 2 (15%) | 4 (36%) | 6 (17%) |
| Don’t know/not applicable | 0 (0%) | 0 (0%) | 0 (0%) | 0 (0%) |
| **solicited opinions of clinical staff regarding decisions about implementation** |  |  |  |  |
| Strongly disagree | 1 (9%) | 1 (8%) | 0 (0%) | 2 (6%) |
| Disagree | 4 (36%) | 2 (15%) | 0 (0%) | 6 (17%) |
| Neither agree nor disagree | 2 (18%) | 3 (23%) | 0 (0%) | 5 (14%) |
| Agree | 2 (18%) | 7 (54%) | 5 (45%) | 14 (40%) |
| Strongly agree | 2 (18%) | 0 (0%) | 6 (55%) | 8 (23%) |
| Don’t know/not applicable | 0 (0%) | 0 (0%) | 0 (0%) | 0 (0%) |
| **Regarding the implementation of telemedicine during the COVID-19 Pandemic as outlined above, the following were available to make the selected plan work...** | | | | |
| **staff incentives** |  |  |  |  |
| Strongly disagree | 9 (82%) | 5 (38%) | 0 (0%) | 14 (40%) |
| Disagree | 2 (18%) | 5 (38%) | 3 (27%) | 10 (29%) |
| Neither agree nor disagree | 0 (0%) | 3 (23%) | 3 (27%) | 6 (17%) |
| Agree | 0 (0%) | 0 (0%) | 4 (36%) | 4 (11%) |
| Strongly agree | 0 (0%) | 0 (0%) | 0 (0%) | 0 (0%) |
| Don’t know/not applicable | 0 (0%) | 0 (0%) | 1 (9%) | 1 (3%) |
| **equipment and materials** |  |  |  |  |
| Strongly disagree | 3 (27%) | 1 (8%) | 0 (0%) | 4 (11%) |
| Disagree | 6 (55%) | 0 (0%) | 0 (0%) | 6 (17%) |
| Neither agree nor disagree | 1 (9%) | 4 (31%) | 2 (18%) | 7 (20%) |
| Agree | 1 (9%) | 7 (54%) | 7 (64%) | 15 (43%) |
| Strongly agree | 0 (0%) | 1 (8%) | 2 (18%) | 3 (9%) |
| Don’t know/not applicable | 0 (0%) | 0 (0%) | 0 (0%) | 0 (0%) |
| **patient awareness or patient need** |  |  |  |  |
| Strongly disagree | 0 (0%) | 0 (0%) | 0 (0%) | 0 (0%) |
| Disagree | 2 (18%) | 0 (0%) | 0 (0%) | 2 (6%) |
| Neither agree nor disagree | 3 (27%) | 3 (23%) | 2 (18%) | 8 (23%) |
| Agree | 3 (27%) | 9 (69%) | 7 (64%) | 19 (54%) |
| Strongly agree | 3 (27%) | 1 (8%) | 2 (18%) | 6 (17%) |
| Don’t know/not applicable | 0 (0%) | 0 (0%) | 0 (0%) | 0 (0%) |
| **provider buy-in** |  |  |  |  |
| Strongly disagree | 1 (9%) | 1 (8%) | 0 (0%) | 2 (6%) |
| Disagree | 1 (9%) | 1 (8%) | 0 (0%) | 2 (6%) |
| Neither agree nor disagree | 2 (18%) | 3 (23%) | 1 (9%) | 6 (17%) |
| Agree | 6 (55%) | 7 (54%) | 7 (64%) | 20 (57%) |
| Strongly agree | 1 (9%) | 1 (8%) | 3 (27%) | 5 (14%) |
| Don’t know/not applicable | 0 (0%) | 0 (0%) | 0 (0%) | 0 (0%) |
| **intervention team** |  |  |  |  |
| Strongly disagree | 0 (0%) | 0 (0%) | 0 (0%) | 0 (0%) |
| Disagree | 7 (64%) | 0 (0%) | 1 (9%) | 8 (24%) |
| Neither agree nor disagree | 3 (27%) | 4 (33%) | 2 (18%) | 9 (26%) |
| Agree | 1 (9%) | 4 (33%) | 6 (55%) | 11 (32%) |
| Strongly agree | 0 (0%) | 2 (17%) | 1 (9%) | 3 (9%) |
| Don’t know/not applicable | 0 (0%) | 2 (17%) | 1 (9%) | 3 (9%) |
| **Regarding the implementation of telemedicine during the COVID-19 Pandemic as outlined above, communication was maintained through...** | | | | |
| **regular project meetings with the project champion (advocate for telemedicine) and team members** |  |  |  |  |
| Strongly disagree | 1 (9%) | 0 (0%) | 0 (0%) | 1 (3%) |
| Disagree | 5 (45%) | 2 (17%) | 0 (0%) | 7 (21%) |
| Neither agree nor disagree | 1 (9%) | 4 (33%) | 1 (9%) | 6 (18%) |
| Agree | 3 (27%) | 4 (33%) | 4 (36%) | 11 (32%) |
| Strongly agree | 1 (9%) | 2 (17%) | 5 (45%) | 8 (24%) |
| Don’t know/not applicable | 0 (0%) | 0 (0%) | 1 (9%) | 1 (3%) |
| **involvement of quality management staff (those who seek to reduce errors/improve patient care) in project planning and implementation** |  |  |  |  |
| Strongly disagree | 3 (27%) | 0 (0%) | 0 (0%) | 3 (9%) |
| Disagree | 6 (55%) | 2 (17%) | 0 (0%) | 8 (24%) |
| Neither agree nor disagree | 1 (9%) | 5 (42%) | 1 (9%) | 7 (21%) |
| Agree | 1 (9%) | 4 (33%) | 6 (55%) | 11 (32%) |
| Strongly agree | 0 (0%) | 0 (0%) | 3 (27%) | 3 (9%) |
| Don’t know/not applicable | 0 (0%) | 1 (8%) | 1 (9%) | 2 (6%) |
| **regular feedback to clinical management staff (i.e. clinic managers, administrators) on progress of project activities and resource needs** |  |  |  |  |
| Strongly disagree | 1 (9%) | 0 (0%) | 0 (0%) | 1 (3%) |
| Disagree | 2 (18%) | 2 (17%) | 0 (0%) | 4 (12%) |
| Neither agree nor disagree | 3 (27%) | 3 (25%) | 0 (0%) | 6 (18%) |
| Agree | 4 (36%) | 5 (42%) | 4 (36%) | 13 (38%) |
| Strongly agree | 1 (9%) | 1 (8%) | 6 (55%) | 8 (24%) |
| Don’t know/not applicable | 0 (0%) | 1 (8%) | 1 (9%) | 2 (6%) |
| **regular feedback to clinicians on effects of practice changes on patient care/outcomes** |  |  |  |  |
| Strongly disagree | 2 (18%) | 1 (8%) | 0 (0%) | 3 (9%) |
| Disagree | 4 (36%) | 3 (25%) | 0 (0%) | 7 (21%) |
| Neither agree nor disagree | 0 (0%) | 2 (17%) | 1 (9%) | 3 (9%) |
| Agree | 4 (36%) | 5 (42%) | 5 (45%) | 14 (41%) |
| Strongly agree | 1 (9%) | 1 (8%) | 4 (36%) | 6 (18%) |
| Don’t know/not applicable | 0 (0%) | 0 (0%) | 1 (9%) | 1 (3%) |
| **Regarding the implementation of telemedicine during the COVID-19 Pandemic as outlined above, the implementation plan for this intervention...** | | | | |
| **identified specific roles and responsibilities** | | | | |
| Strongly disagree | 1 (9%) | 0 (0%) | 0 (0%) | 1 (3%) |
| Disagree | 4 (36%) | 0 (0%) | 0 (0%) | 4 (11%) |
| Neither agree nor disagree | 3 (27%) | 3 (23%) | 1 (9%) | 7 (20%) |
| Agree | 3 (27%) | 9 (69%) | 5 (45%) | 17 (49%) |
| Strongly agree | 0 (0%) | 1 (8%) | 5 (45%) | 6 (17%) |
| Don’t know/not applicable | 0 (0%) | 0 (0%) | 0 (0%) | 0 (0%) |
| **clearly described tasks and timelines** |  |  |  |  |
| Strongly disagree | 1 (9%) | 0 (0%) | 0 (0%) | 1 (3%) |
| Disagree | 3 (27%) | 0 (0%) | 0 (0%) | 3 (9%) |
| Neither agree nor disagree | 5 (45%) | 4 (31%) | 1 (9%) | 10 (29%) |
| Agree | 2 (18%) | 8 (62%) | 5 (45%) | 15 (43%) |
| Strongly agree | 0 (0%) | 1 (8%) | 5 (45%) | 6 (17%) |
| Don’t know/not applicable | 0 (0%) | 0 (0%) | 0 (0%) | 0 (0%) |
| **included appropriate provider/patient education** |  |  |  |  |
| Strongly disagree | 1 (9%) | 0 (0%) | 0 (0%) | 1 (3%) |
| Disagree | 4 (36%) | 0 (0%) | 0 (0%) | 4 (11%) |
| Neither agree nor disagree | 4 (36%) | 1 (8%) | 1 (9%) | 6 (17%) |
| Agree | 2 (18%) | 11 (85%) | 5 (45%) | 18 (51%) |
| Strongly agree | 0 (0%) | 1 (8%) | 5 (45%) | 6 (17%) |
| Don’t know/not applicable | 0 (0%) | 0 (0%) | 0 (0%) | 0 (0%) |
| **acknowledged staff input and opinions** |  |  |  |  |
| Strongly disagree | 1 (10%) | 0 (0%) | 0 (0%) | 1 (3%) |
| Disagree | 3 (30%) | 1 (8%) | 0 (0%) | 4 (12%) |
| Neither agree nor disagree | 3 (30%) | 5 (38%) | 2 (18%) | 10 (29%) |
| Agree | 3 (30%) | 6 (46%) | 3 (27%) | 12 (35%) |
| Strongly agree | 0 (0%) | 1 (8%) | 6 (55%) | 7 (21%) |
| Don’t know/not applicable | 0 (0%) | 0 (0%) | 0 (0%) | 0 (0%) |

Legend: This table details the self-reported use of these implementation strategies during the initial implementation of teledermatology during the COVID-19 pandemic by respondents from each ORCA tertile.
